# Efficacy and safety of tofacitinib combined with methotrexate in the treatment of rheumatoid arthritis: A systematic review and meta-analysis

**DOI:** 10.1016/j.heliyon.2023.e15839

**Published:** 2023-04-28

**Authors:** Yan Gao, Yi-ni Gao, Mei-jiao Wang, Yi Zhang, Feng-qi Zhang, Zhi-xing He, Wu Chen, Hai-chang Li, Zhi-jun Xie, Cheng-ping Wen

**Affiliations:** Key Laboratory of Chinese Medicine Rheumatology of Zhejiang Province, School of Basic Medical Sciences, Zhejiang Chinese Medical University, Hangzhou, China

**Keywords:** Tofacitinib, Methotrexate, Rheumatoid arthritis, Randomized controlled trial, Meta-analysis, Systematic review

## Abstract

**Objective:**

To evaluate the efficacy and safety of tofacitinib in combination with methotrexate (MTX) versus MTX monotherapy in patients with active rheumatoid arthritis (RA).

**Methods:**

Trials were identified from four electronic databases: PubMed, Web of science, Cochrane Library and EMBASE from inception to April 2022. Two independent reviewers evaluated each database to scan the title, abstract and keywords of each record retrieved. Full articles were further assessed when the information suggested that the study was a randomized clinical trial (RCT) comparing tofacitinib combined with MTX vs. MTX monotherapy in patients with active RA. Data were extracted from the literature, and the methodological quality of the included literature were evaluated and screened by two reviewers independently. The results were analyzed using RevMan5.3 software. The full text of the studies and extracted data were reviewed independently according to PRISMA guidelines. The outcome indicators were ACR 20, ACR 50, ACR 70, Disease activity score 28 (DAS28), erythrocyte sedimentation Rate (ESR) and adverse events (AEs).

**Results:**

Of 1152 studies yielded by the search, 4 were retained, totaling 1782 patients (1345 treated with tofacitinib combined with MTX vs 437 received MTX. In the trial of insufficient response to MTX treatment, tofacitinib combined with MTX had significant advantages compared with MTX monotherapy. Numerically higher ACR20, ACR50 and ACR70 response rates were observed in the tofacitinib combined with MTX groups versus MTX monotherapy. ACR20 (odds ratio (OR), 3.62; 95% CI, 2.84–4.61; *P* < 0.001), ACR50 (OR, 5.17; 95% CI, 3.62–7.38; *P* < 0.001), and ACR70 (OR, 8.44; 95% CI, 4.34–16.41; *P* < 0.001), DAS28 (ESR) < 2.6 (OR, 4.71, 95% CI, 2.06–10.77; *P* < 0.001). The probability of adverse events of tofacitinib combined with MTX was lower than that of MTX monotherapy (OR, 1.42; 95% CI, 1.08–1.88; *P* = 0.01). The number of cases discontinued due to lack of efficacy or adverse events was similar in both groups (OR, 0.93; 95% CI, 0.52–1.68). The probability of abnormal liver enzymes in the treatment of tofacitinib combined with MTX was significantly lower than that of MTX monotherapy (OR, 1.86; 95% CI, 1.35–2.56). However, there was no significant difference between the two groups in severe adverse reactions, neutropenia, anemia and cardiovascular disease.

**Conclusions:**

In terms of ACR20/50/70 and DAS28 (ESR), tofacitinib combined with MTX demonstrated superiority to MTX monotherapy in the treatment of patients with refractory RA. Considering the hepatoprotective and observably therapeutic efficacy, tofacitinib combined with MTX could be effective in treating refractory RA. However, in terms of hepatoprotective, it requires further large-scale and high-quality clinical trials to confirm.

## 1 Introduction

Rheumatoid arthritis (RA) is one of the most common chronic aggressive autoimmune diseases in the world and is characterized by inflammation of the synovium, hyperplasia, production of autoantibody, destruction of the cartilage and joint [[1], [2], [3]]. RA causes joint deformities, which leading patients to lose their ability to live and even disability [4]. Therefore, early diagnosis and active treatment are the basic strategies to inhibit inflammation before irreversible injury [5]. In order to alleviate symptoms and cut down mortality rate, the majority of patients receive disease-modifying anti-rheumatic drugs (DMARDs) permanently [6].

Methotrexate (MTX), as a conventional synthetic DMARDs (csDMARDs), is still the cornerstone of the treatment of RA. Some patients can benefit from csDMARDs treatment. It can achieve immunosuppressive and anti-inflammatory effects by inhibiting the synthesis of purine and pyrimidine, thus effectively relieving the symptoms and pain of RA patients. However, it is effective in only 19.8–25.4% of RA patients [7]. In addition, the side effects of MTX are also significant, including myelosuppression [8], gastrointestinal disorders [9], hepatotoxicity [[9], [10], [11], [12]], pulmonary damage, dermatitis, and so on [13]. Therefore, a novel strategy to overcome the dilemma phenomenon of MTX is expected clinically.

Tofacitinib is a new small-molecule inhibitor of several JAK subtypes, especially JAK3 and JAK1. Tofacitinib acts on synovial JAK/STAT targets through JAK mediated IFN and IL-6 signaling pathways, thus blocking the role of JAK in synovial response to play a therapeutic role in RA [14]. Studies have shown that tofacitinib can not only effectively relieve RA patients with poor MTX response [[15], [16], [17], [18]], but also reduce the risk of major adverse cardiovascular events (MACE) in RA patients [19,20].

Currently, for patients with inadequate response (IR) to MTX, biologic DMARDs (bDMARDs), such as tumor necrosis factor inhibitors (TNFi), or targeted synthetic small-molecule DMARDs (tsDMARDs), are usually used in combination with MTX [[21], [22], [23]]. Furthermore, in the European Union, tofacitinib is recommended for the treatment of moderate to severe active RA in adult patients with inadequate or intolerable response to one or more DMARDs [24]. So far, several clinical studies have demonstrated that tofacitinib combined with MTX can effectively improve the symptoms of patients with insufficient response to MTX [17,18,25]. However, high-quality evidence-based meta-analysis data on whether tofacitinib combined with MTX is superior to MTX monotherapy are not available.

To assess the clinical efficacy and safety of tofacitinib combination with MTX, we conducted this meta-analysis and examined therapy -related adverse events (AEs), and tried to find further evidence for the clinical application of tofacitinib combination with MTX treatment resistant RA patients.

## 2 Methods

This meta-analysis was conducted according to the Preferred Reporting Items for Systematic Review and Meta-Analysis statement [26], and a prior protocol for this study was registered at the International Prospective Register of Systematic Reviews (PROSPERO: CRD42022353885 URL: https://www.crd.york.ac.uk/prospero/#recordDetails).

### 2.1 Literature search

Data searches were conducted in electronic database PubMed, Web of Science, Cochrane Library and Embase until April 2022, with no start date specified. Several words including “Rheumatoid Arthritis”,“methotrexate”, “tofacitinib” and “randomized controlled trial” were used as Medical Subject Headings (MeSH) terms to identify all articles on the combination of tofacitinib and MTX for the treatment of RA. Language were not limited during the search.

### 2.2 Study selection

1) Inclusion criteria: Trials that meet all of the following criteria were considered eligible for inclusion: (i) All patients fulfilled the criteria revised by the American College of Rheumatology in 1987, (ii) Types of studies: Randomized controlled trials (RCTs) comparing MTX monotherapy with tofacitinib in combination with MTX lasted at least 12 weeks. (iii) Types of participants: Adult RA patients at 18 years old or above. (iv) Methods of interventions: MTX combination with tofacitinib as the intervention group, and MTX monotherapy or MTX plus placebo as the control group. Simultaneous use of glucocorticoids was permitted.
2) Exclusion criteria: Trials without RCT designs, without results of interest were excluded, as were trials without MTX or MTX combined with placebo as a control group.

### 2.3 Data extraction

Two independent reviewers (Yan Gao and Yini Gao) screened the titles and abstracts of the citations and retrieved relevant articles based on pre‐specified inclusion and exclusion criteria. Two reviewers (Meijiao Wang, Yi Zhang) independently extracted the data including name of first author, time of publication, sampling size, duration of therapy, dose and results. The Cochrane Collaboration Risk of Bias Tool was used by two reviewers (Yan Gao and Fengqi Zhang) independently to conduct the methodological quality assessment [27]. Discrepancies were resolved through discussion.

### 2.4 Types of outcome measures

1) Efficacy: The efficacy of any of the following: ACR20/50/70, DAS28 (ESR) [5]. 2) Safety: Frequency of patients who experienced total AEs or individual AEs such as hepatotoxicity (transaminitis), hematological AEs (anemia, leucopenia, and/or thrombocytopenia), and cardiovascular events.

### 2.5 Analysis of statistic

Data was analyzed by software of Review Manager 5.3 (Cochrane Collaboration, Oxford, UK) Cochrane Collaboration's tool for assessing the risk of bias (RoB2) [28] was used for methodological quality assessment. Odds ratios (OR) of dichotomous data were calculated with 95% confidence intervals (CIs). If there was statistical heterogeneity between studies, a random effect model was employed to estimate of the incidence for each outcome; If not, the fixed effect model was employed [29]. Assessment of heterogeneity used Cochrane's chi-square test, and the degree of inconsistency between studies was measured by Higgins *I*^*2*^. When the value was 0%, it indicated that no heterogeneity was observed. Higher values indicate greater heterogeneity. A value greater than 50% may indicate that there is substantial heterogeneity [30]. Statistical significance was set at *P* < 0.05.

## 3 Results

### 3.1 Study selection and characteristics

**Fig. 1:**
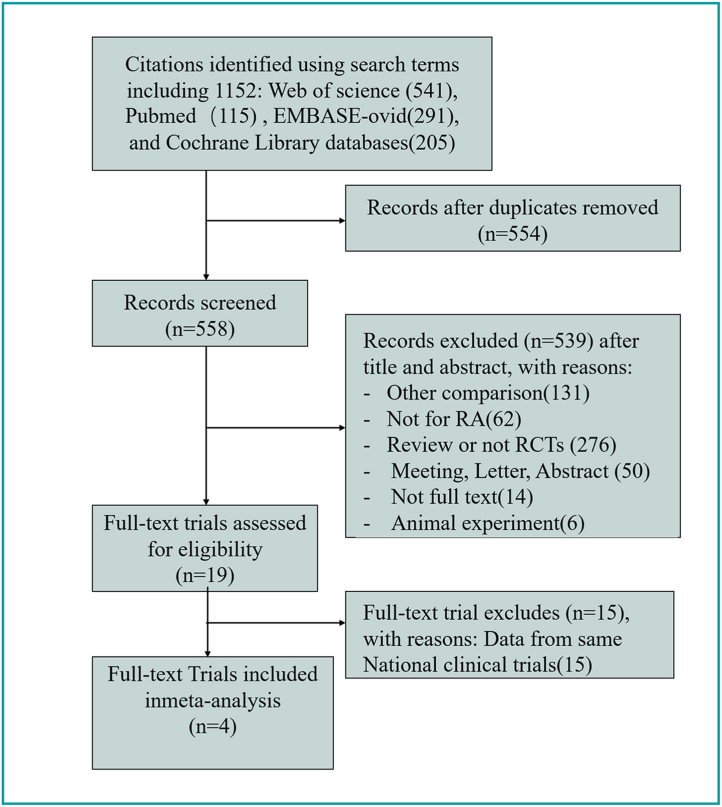
Flow chart of study selection. RA = Rheumatoid arthritis; RCT = randomized controlled trial.

Employing the abovementioned search strategy, we retrieved 1152 potentially relevant articles. After the removal of duplicates, 558 studies were screened. The content review limited the relevant papers to four studies that met the inclusion criteria for the meta-analysis. The general procedure for study selection is shown in Fig. 1.

A total of four trials including 1345 tofacitinib + MTX cases and 437 MTX, met our inclusion criteria and were included in the present study. The tofacitinib doses ranged between 5 and 10 mg twice daily with 5 mg twice daily in chief. MTX dose ranged from 7.5 to 25 mg/week were used in combination or monotherapy. The duration of the interventions varied from 12 to 48 weeks and the effectiveness and safety of therapy assessed by five types of results such as ACR20/50/70, DAS28 (ESR) < 2.6 and AEs. The characteristics of the included RCTs are summarized in Table 1.

**Table 1:** Characteristics of the four trials included in the meta-analysis.

Author	Year	Region	Trial Identifier	Sample size（female/male）		Intervention		Mean age（years）		Disease duration（years）		DAS28-4 (ESR), mean (SD)		Duration (weeks)
				Trial group	Control group	Trial group	Control group	Trial group	Control group	Trial group	Control group	Trial group	Control group	
Philip et al.	2016	Latin America, Europe USA	NCT01164579	31/5	29/8	36	37	47.8 ± 12.3	47.8 ± 11.6	0.8 ± 0.52	0.6 ± 0.45	6.3 ± 0.9	6.4 ± 0.8	48
De′sire′e et al.	2013	North America, South America, Europe, Asia, Australia	NCT00847613	541/96	137/23	637	160	52.8 ± 11.5	52.6 ± 11.6	9.1 ± 9.2	9.1 ± 8.9	6.29	6.27	12
Gerd et al.	2013	North America, Europe, and Latin America	NCT00960440	229//38	106/26	267	132	55.2 ± 11.3	54·4 ± 11.3	12.8 ± 11.9	11.3 ± 10.75	6.4 ± 1.0	6.4 ± 1.1	12
Ronald et al.	2012	North America Latin America Europe Rest of world	NCT00853385	342/63	82/26	405	108	52.9 ± 11.8	53.8 ± 13.7	7.95	7.5	72/108	6.6	12

### 3.2 Determination of bias

**Fig. 2:**
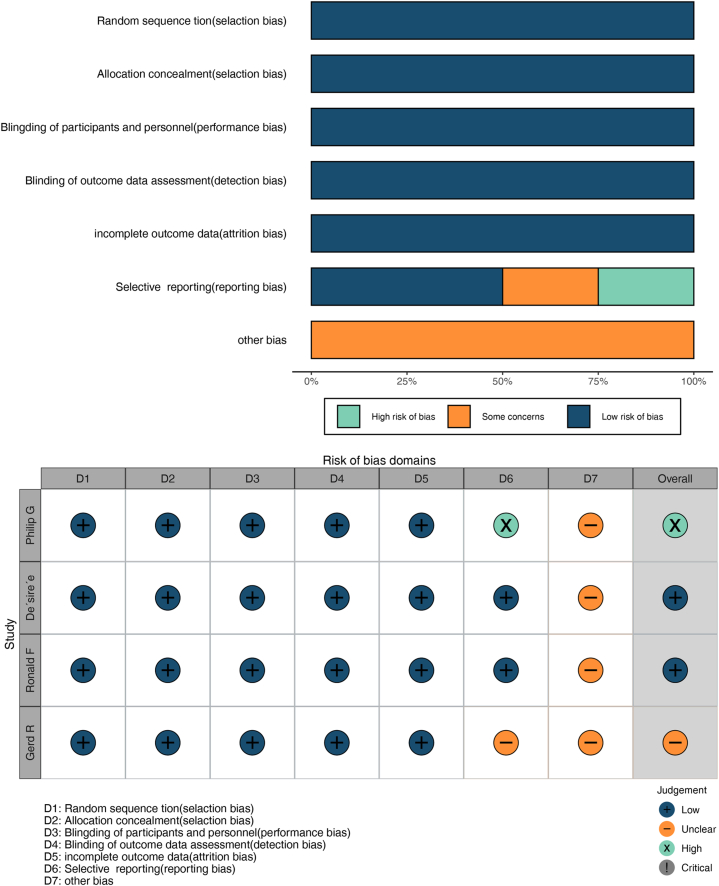
Risk-of-bias summary.

The risk of bias was low for the four studies included in the meta-analysis (Fig. 2). All trials were blinded including participants and investigators. All trials described the method of randomization and concealment of allocation.

### 3.3 The efficacy and safety of tofacitinib with MTX vs MTX monotherapy

**Fig. 3:**
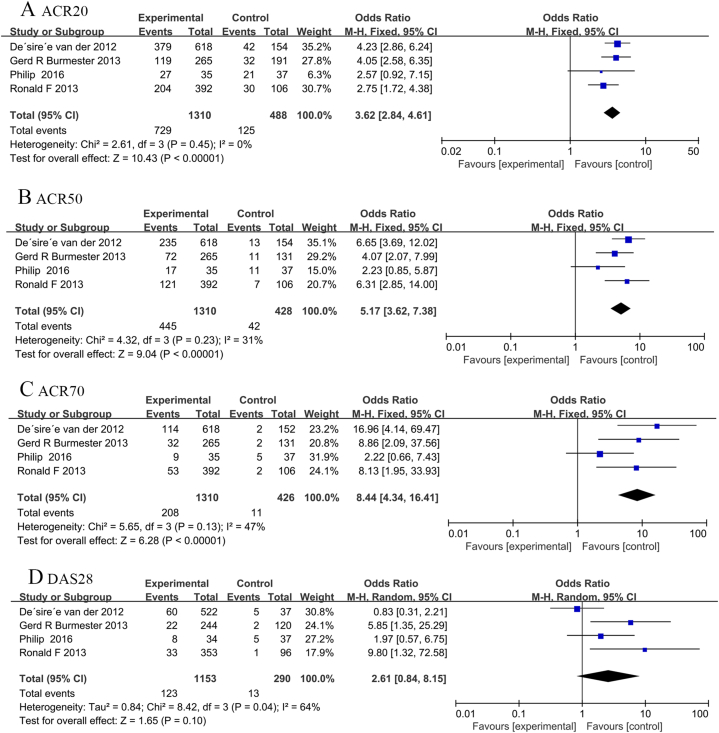
Pooled effects of Efficacy and safety of MTX combined with tofacitinib vs. MTX monotherapy for RA. Forest plots comparing tofacitinib plus MTX and MTX monotherapy for RA. (A) ACR20; (B) ACR50; (C) ACR70; （D）DAS28; MTX, methotrexate; ACR20, American college of Rheumatology 20; ACR50, American college of Rheumatology 50; ACR70, American college of Rheumatology 70; DAS28, Disease Activity Score.

ACR criteria (ACR20/50/70) and DAS28 (ESR) < 2.6 were used for efficacy evaluation. In conclusion, tofacitinib combined with MTX can effectively alleviate disease activity. Summary analysis demonstrated a higher ACR20/50/70 response rate and low heterogeneity for tofacitinib combination with MTX compared with MTX monotherapy (ACR20:OR, 3.62; 95% CI, 2.84–4.61; *P* < 0.001; heterogeneity: *P* = 0.45; *I*^*2*^ = 0%; Fig. 3A; ACR50: OR, 5.17; 95% CI, 3.62–7.38; *P* < 0.001; heterogeneity: *P* = 0.23; *I*^*2*^ = 31%; Fig. 3B; ACR70: OR, 8.44; 95% CI, 4.34 to 16.41; *P* < 0.001; heterogeneity: *P* = 0.13; *I*^*2*^ = 47%; Fig. 3C) As shown in Fig. 3D, according to the fixed effect model (*I*^*2*^ = 64%; *P* = 0.04), four studies showed DAS28 (ESR) < 2.6 and the pooled results revealed a significantly lower rate of DAS28 (ESR) < 2.6 caused by MTX combined with tofacitinib treatment (OR = 2.68; 95% CI, 1.44–4.99; *P* = 0.0002). Due to high heterogeneity, sensitivity analysis was performed after eliminating the study by Gerd R et al., and the heterogeneity was significantly reduced (*I*^*2*^ reducing from 64% to 21%; Fig. 5). Subgroup analysis of DAS28 < 2.6 (ESR) showed that the efficacy of tofacitinib 10 mg plus MTX was no difference with tofacitinib 5 mg plus MTX. (OR = 4.99; 95% CI, 1.92–12.97; *P* = 0.96; OR = 7.13; 95% CI, 3.51–14.48; *P* = 0.16; respectively) (Fig. 6).

### 3.4 AEs

**Fig. 4:**
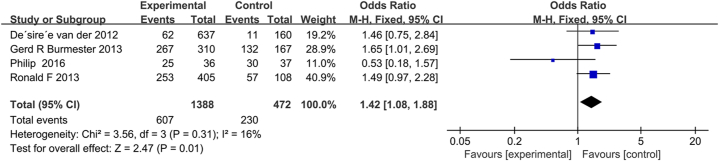
Pooled effects of total AEs of MTX combined with tofacitinib vs. MTX monotherapy for RA. Forest plots comparing MTX and MTX plus tofacitinib treatment. Abbreviations: AEs, adverse events; MTX, methotrexate; RA, rheumatoid arthritis.

AEs resulting from tofacitinib in combination with MTX or MTX monotherapy were reported in four studies. No heterogeneity among the above studies was found using fixed effects model analysis (*P* = 0.31 and *I*^*2*^ = 16%). As the results in Fig. 4 showed that the AEs resulting from tofacitinib combination with MTX were significantly lower than those caused by MTX alone (*P* = 0.01; 95% CI, 1.08–1.88). Four trials [17,[31], [32], [33]] reported serious adverse events and cardiovascular, three trials [17,31,32] reported abnormal liver function, hemoglobin reduction and drug withdrawal due to AEs, two trials [31,32] reported neutropenia. The subgroup analysis results of AEs shown in Fig. 7 indicate that the incidence of abnormal liver function in tofacitinib combined with MTX was significantly lower than that in MTX monotherapy (OR, 1.86; 95% CI, 1.35–2.56; *P* = 0.0001); However, there was no significant difference between the two groups in severe adverse reactions (OR,1.03; 95%CI, 0.57–1.87; *P* = 0.92), neutropenia (OR, 1.06, 95% CI, 0.56–2.03; *P* = 0.86), hemoglobin reduction (OR = 0.97; 95% CI, 0.59–1.58; *P* = 0.89) and cardiovascular disease (OR, 2.08; 95% CI, 0.76–5.70; *P* = 0.16), and the two groups had similar OR due to the lack of efficacy or AEs discontinuations. (OR = 0.93; 95% CI, 0.52–1.68; *P* = 0.82).

**Fig. 5:**
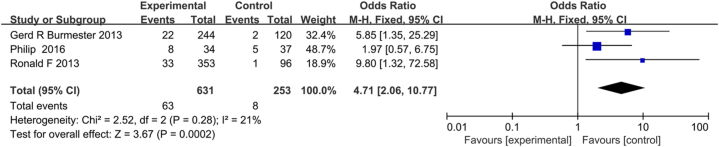
The sensitivity analysis of DAS28 Abbreviations: DAS28, Disease Activity Score.

**Fig. 6:**
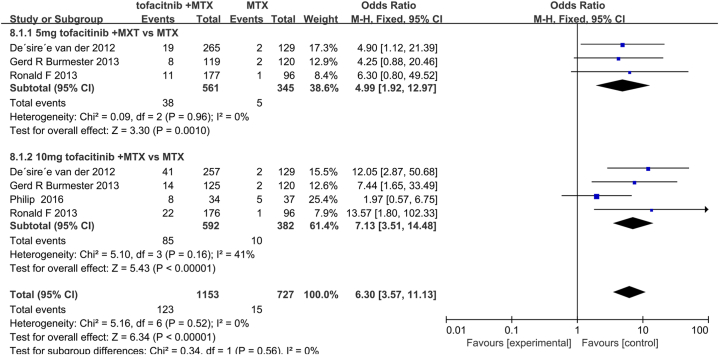
Subgroup analysis of DAS28 based on different doses of tofacitinib. Forest plots comparing tofacitinib plus MTX and MTX monotherapy for RA. Abbreviations: MTX, methotrexate; DAS28, Disease Activity Score; RA, rheumatoid arthritis.

**Fig. 7:**
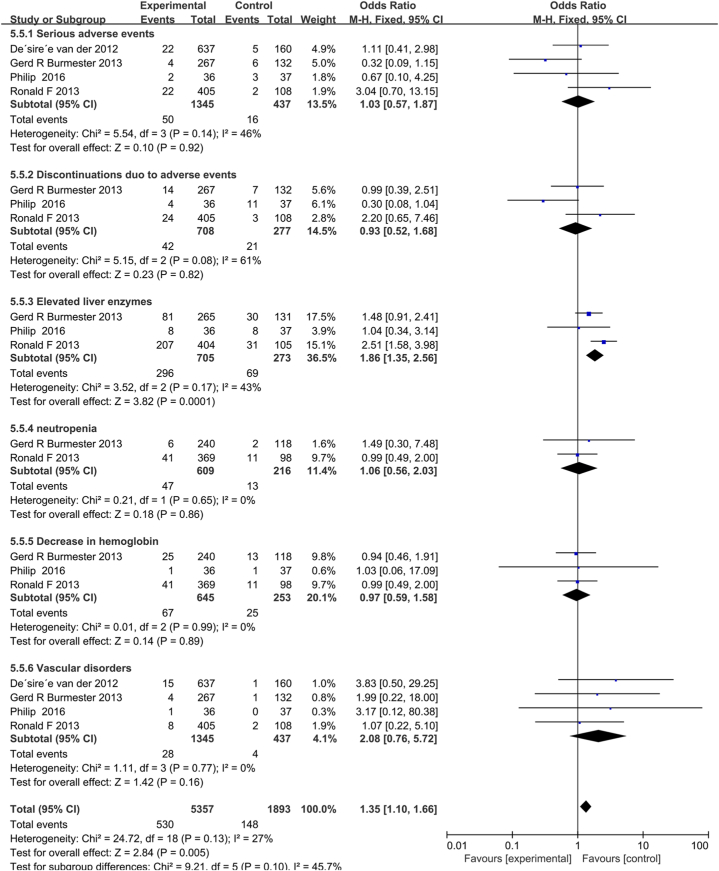
Subgroup analysis of AEs based on follow-up. Forest plots comparing tofacitinib plus MTX and MTX monotherapy for RA. Abbreviations: AEs, adverse events; MTX, methotrexate; RA, rheumatoid arthritis.

## 4 Discussion

This review included four trials with 1782 patients. We applied a rigorous method to identify trials and abstraction of outcomes. Our pooled results indicated that MTX combination with tofacitinib was better than oral MTX monotherapy for ACR20/50/70 response and DAS28 [ESR]＜2.6, in MTX inadequate response population. ACR20 (OR, 3.62; 95% CI, 2.84–4.61; *P* < 0.001), ACR50 (OR, 5.17; 95% CI, 3.62–7.38; *P* < 0.001), ACR70 (OR, 8.44; 95% CI, 4.34–16.41; *P* < 0.001), and DAS28 [ESR]＜2.6 (*P* = 0.00; *I*^*2*^ = 64%). In addition, this review determined those AEs of MTX combined with tofacitinib in the treatment of RA were significantly lower than those of MTX monotherapy (*P* = 0.01; 95% CI, 1.08–1.88). In summary, this meta-analysis provides valuable insights into the efficacy and safety of MTX combination with tofacitinib intreating refractory RA, and is helpful for doctors and patients to discuss issues before starting to use tofacitinib combined with MTX.

### 4.1 Reliability of statistical conclusion

ACR20, ACR50 [34] and ACR70 [35] have been conventionally used to evaluate the degree of disease remission. DAS28 [36] has good predictive value and has become an important indicator for evaluating the disease activity of RA for years. Interestingly, based on RA clinical trials reported since 1997, ACR20 and ACR50 have been found to have similar efficacy in assessing disease remission [34]. Therefore, ACR20, ACR50, ACR70 and DAS28 can reflect the effectiveness of drug therapy for RA [37].

The efficacy of tofacitinib combined with MTX in treating RA can be explained by some mechanism studies. Firstly, MTX not only alleviates inflammation by significantly reducing proinflammatory cytokines (IFN- γ, TNF-α, IL-1β, IL-6, IL-15 and IL-18) [[38], [39], [40]], but also delays disease progression by regulating the level of joint destroying enzymes such as iNOS, NOS2 and COX-2 [40]. Secondly, tofacitinib as a targeted small molecule inhibitor of several JAK subtypes, acts on synovial JAK/STAT targets through IFN and IL-6 signaling pathways, thus blocking the role of JAK in synovial response to play a therapeutic role in RA [14,17,18]. Most of all, on the part of combined treatment, multiple RCTs showed that tofacitinib in combination with MTX was superior to MTX monotherapy in treating various efficacy endpoints, including ACR response criteria and DAS28 [[31], [32], [33]].

Unfortunately, patients compliance with MTX during clinical implementation was relatively low. A study showed that patients often stop taking it due to hepatotoxicity [10] which affected their compliance with MTX. Among RA patients starting MTX, there were a lot of side-effects, including bone marrow restrain [8], gastrointestinal disturbances, liver toxicity [10], lung injury, dermatitis, and so on [13]. Therefore, high-quality evidence is urgently needed to clarify whether tofacitinib combined with MTX treatment can replace the MTX monotherapy, so as to avoid insufficient disease control of some RA patients under MTX monotherapy.

Notably, tofacitinib combined with MTX resulted in fewer AEs than MTX monotherapy, especially when hepatotoxicity was highlighted. The result would benefits patients who cannot tolerate monotherapy with MTX.

The subgroup analysis of DAS28 < 2.6 (ESR) showed that the efficacy of tofacitinib with MTX was independent of the dose of tofacitinib. One possible reason is that data from RCT at various doses are insufficient to support this conclusion. Another reason is false positive or false negative results due to the limited number of literature included in the study. In addition, various doses of MTX and tofacitinib may lead to diversity and complexity of combined treatment. High-quality dose combinations should be conducted for various additional tests to determine the best treatment scheme. Relevant results with DAS28 < 2.6 (ESR) should be noted, taking into account the variation in sensitivity analyses.

### 4.2 Highlights and limitations

The present study had many advantages. As far as we know, this was the first large-scale, up-to-date or comprehensive meta-analysis of this issue. We used a rigorous method to t identify trials and abstraction of results, so we are confident that the results contain the best evidence from RCT showing that the therapeutic effects of tofacitinib combination with MTX was better than MTX monotherapy. As Fig. 3 shown, the effect of MTX combination with tofacitinib on ACR20/50/70, DAS28 [ESR] < 2.6 was better than those of MTX monotherapy. Furthermore, the pooled results suggest those AEs induced by tofacitinib in combination with MTX were significantly different from those induced by MTX alone.

The interpretation of data is challenging because there are several weaknesses in the results of systematic literature retrieval. Firstly, these studies are heterogeneous in terms of DAS28. In addition, the dose of tofacitinib in clinical trials was not always consistent. Some trials used 5 mg twice daily, while others used 10 mg twice daily. This study shows that the efficacy of tofacitinib with MTX was independent of the dose. Finally, the patient population included in the study came from a variety of continents. Most patients came from North America, South America or Europe. The duration of treatment was limited to 3–12 months as these patients were severely refractory RA. At the same time, the imaging progress of RA patients was not evaluated. Therefore, longer trials and more data are needed to conclusively support the conclusion that MTX in combination with tofacitinib is effective and safe in the long term.

## 5 Conclusion

In summary, based on all the available evidence in this treatment-refractory population, our results show that MTX with tofacitinib is effective for patients with insufficient MTX response, and there is a significant statistical difference between MTX plus tofacitinib and MTX alone in controlling disease activity. MTX with tofacitinib may provide an effective option in patients who do not respond adequately to MTX. therapy.

## Author contribution statement

All authors listed have significantly contributed to the development and the writing of this article.

## Data availability statement

Data included in article/supplementary material/referenced in article.

## Additional information

No additional information is available for this paper.

## Funding information

This study is financially supported by the Zhejiang Province TCM Science and Technology Program (Grant NO. 2020ZZ006), 10.13039/501100004731Zhejiang Provincial Natural Science Foundation of China (Grant NO. Y21H270014), and 10.13039/501100012166National Key R&D Program of China (Grant NO·2018YFC1705500).

## Ethical statement

The authors are accountable for all aspects of the work in ensuring that questions related to the accuracy or integrity of any part of the work are appropriately investigated and resolved. The present study was registered and approved by PROSPERO under the registration number of CRD42022353885.

## Declaration of competing interest

The authors declare that they have no competing interests.
